
JOURNAL INFORMATION
==============================
NLM Title Abbreviation: Wirtschaftsdienst
Journal ID: Wirtschaftsdienst
NLM Journal ID: 101086612

Wirtschaftsdienst (Hamburg, Germany : 1949)

ISSN: 0043-6275

ARTICLE INFORMATION
==============================
PMCID: PMC7368599
PMID: 32834160
DOI: 10.1007/s10273-020-2683-6
Article ID: 2683
Article version: 1
Subjects: Leitartikel

Mehr europäische Projekte im Wiederaufbauprogramm!

More European projects in the recovery programme

Watt Andrew Andrew-Watt@BOECKLER.DE
1
Holzner Mario holzner@wiiw.ac.at
2
1 in der Hans-Böckler-Stiftung: Europäische Wirtschaftspolitik, Inst. f. Makroökonomie u. Konjunkturf. (IMK), Georg-Glock-Str. 18, 40474 Düsseldorf, Deutschland
2 grid.426374.0 0000 0001 0806 9449 Wirtschaftsvergleiche (wiiw), Wiener Institut für Internationale, Rahlgasse 3, 1060 Wien, Österreich
Electronic publication date: 2020 Jul 18
Print publication date: 2020
Volume: 100
Issue: 7
First page: 474
Last page: 475
Copyright: © Der/die Autor(en) 2020
License: Open Access Dieser Artikel wird unter der Creative Commons Namensnennung 4.0 International Lizenz veröffentlicht, welche die Nutzung, Vervielfältigung, Bearbeitung, Verbreitung und Wiedergabe in jeglichem Medium und Format erlaubt, sofern Sie den/die ursprünglichen Autor(en) und die Quelle ordnungsgemäß nennen, einen Link zur Creative Commons Lizenz beifügen und angeben, ob Änderungen vorgenommen wurden.
Die in diesem Artikel enthaltenen Bilder und sonstiges Drittmaterial unterliegen ebenfalls der genannten Creative Commons Lizenz, sofern sich aus der Abbildungslegende nichts anderes ergibt. Sofern das betreffende Material nicht unter der genannten Creative Commons Lizenz steht und die betreffende Handlung nicht nach gesetzlichen Vorschriften erlaubt ist, ist für die oben aufgeführten Weiterverwendungen des Materials die Einwilligung des jeweiligen Rechteinhabers einzuholen.
Weitere Details zur Lizenz entnehmen Sie bitte der Lizenzinformation auf http://creativecommons.org/licenses/by/4.0/deed.de.
License URL: https://creativecommons.org/licenses/by/4.0/

issue-copyright-statement: © The Author(s) 2020

==============================

Deutschland übernimmt die sechsmonatige Präsidentschaft der EU in bewegten Zeiten. Zwei deutsche Politikerinnen, Bundeskanzlerin Merkel und Kommissionspräsidentin von der Leyen, stehen auf der Brücke und müssen den Tanker Europäische Union in schwierigen Gewässern steuern. Zu den üblichen Daueraufgaben der europäischen Politik gesellen sich neue Herausforderungen, die an – mindestens – drei miteinander verlinkten Fronten auf europäische Lösungen drängen: Die rasche Erholung von dem tiefen durch die Pandemie verursachten wirtschaftlichen Einbruch; die immer dringlicher erscheinende Dekarbonisierung; und die angesichts der Entwicklungen in den USA, China und anderen mächtigen Staaten immer klarere Erkenntnis, dass Europa eine größere strategische Autonomie anstreben muss.

Die gute Nachricht: Es ist in den letzten Monaten schon erstaunlich viel in Bewegung geraten, alte Tabus scheinen gebrochen und, in manchen Bereichen zumindest, wurden konkrete politische Entscheidungen getroffen. Die EZB hat entschlossen reagiert: das Krisenprogramm Pandemic Emergency Purchase Programme (PEPP) macht rasch und zielgerichtet zusätzliche Käufe öffentlicher Anleihen der Mitgliedstaaten möglich. Die EU-Kommission hat die Fiskalregeln und das Regelwerk für Staatsbeihilfen temporär auf Eis gelegt, um die Anti-Krisenpolitik der Mitgliedstaaten nicht zu konterkarieren. Mit dem SURE (Support to mitigate Unemployment Risks in an Emergency)-Instrument zur Unterstützung nationalstaatlicher Kurzarbeitsprogramme und der Einrichtung einer neuen Kreditlinie des Europäischen Stabilitätsmechanismus (bis zu 2 % des BIP) bei geringer Konditionalität wird eine konkrete finanzielle Unterstützung geleistet. Dies bleibt aber weit unter der Schwelle dessen, was nötig ist. Zumindest solange die EZB am PEPP-Programm festhält, ist der finanzielle Vorteil weiterer verbilligter Darlehen gering. Umso wichtiger ist daher der Wiederaufbauplan der EU-Kommission unter dem marketingtechnisch eher ungelungenen Titel „Next Generation EU“. Mit seinem Volumen und der Aufnahme gemeinsamer langfristiger Anleihen, die über den (erweiterten) EU-Haushalt bedient werden, markiert der Plan einen grundlegenden Wandel in der europäischen Integration. Damit steht oder fällt die deutsche Präsidentschaft mit der Frage, ob der Plan annähernd in der vorgeschlagenen Form – gegenwärtig sind Zuweisungen von 500 Mrd. Euro, Darlehen von 250 Mrd. Euro vorgesehen – trotz der Opposition aus Ländern wie den Niederlanden und Österreich umgesetzt wird.

Es ist nur ein scheinbarer Widerspruch, die historische Bedeutung des Next-Generation-EU-Ausbauplans zu betonen und festzustellen: Auch wenn er mehr oder minder wie geplant umgesetzt wird, reicht er nicht aus. Das liegt einerseits daran, dass die EU in den vergangenen Jahren nur ansatzweise die massiven Defizite der Economic Governance, die von der Finanzkrise erbarmungslos offenbart wurden, aufgearbeitet hat. Und andererseits an den schon erwähnten drei ineinandergreifenden Herausforderungen. Das ist nicht nur eine Frage des finanziellen Volumens. Es geht um das Umsetzungsprinzip: europäisches Geld für nationale Projekte. Selbst wenn hier eine gewisse Koordination über nationale Umsetzungspläne erfolgen soll – solche „Konditionalität“ ist einer der vielen Zankäpfel in den Verhandlungen – ist es alles andere als sicher, dass die Summe der nationalen Aktionen das Optimale für Europa insgesamt herausholt. Was ist gewonnen, wenn 27 Länder ihre jeweilige Zukunftsvision einer grünen

Wasserstoff-Wirtschaft zu verwirklichen versuchen? Daher sind neben europäischer Unterstützung für nationale Prioritäten genuin europäische Projekte, eine gemeinsame Investition in europäische öffentliche Güter vonnöten. In einer gemeinsamen Policy Note schlagen das Institut für Makroökonomie und Konjunkturforschung (IMK) in Düsseldorf, das Observatoire français des conjonctures économiques (OFCE) in Paris und das Wiener Institut für Internationale Wirtschaftsvergleiche (wiiw) ein auf zehn Jahre angelegtes Investitionsprogramm in Höhe von 2 Billionen Euro vor, das sich auf die Bereiche öffentliche Gesundheit, Verkehrsinfrastruktur und Energie/Dekarbonisierung konzentriert. Dieses würde gesamteuropäische Projekte finanzieren, damit die EU gestärkt aus der COVID-19-Krise hervorgehen kann. Es besteht aus zwei Säulen.

In einer nationalen Säule würden den Mitgliedstaaten – im Großen und Ganzen wie im Kommissionsvorschlag – 500 Mrd. Euro zugewiesen. Die Ressourcen sollten auf die am stärksten betroffenen Länder konzentriert und zeitlich vorgezogen werden. Der größte Teil des Geldes – 1,5 Billionen Euro – wäre für die Finanzierung europäischer Projekte bestimmt, bei denen ein sichtbarer EU-Mehrwert entsteht. Allen Projekten gemeinsam sollte sein, dass sie geeignet sind, möglichst allen Zielen zu dienen: Die kurzfristige Erholung stärken, Produktivität und damit Lebensstandards erhöhen, die regionale Kohäsion befördern, die Dekarbonisierung beschleunigen und die globale strategische Position der EU stärken. Initiativen, die die EU in den Bereichen öffentliche Gesundheit, Verkehrsinfrastruktur und Energie/Dekarbonisierung ergreifen könnten, wären z.B. die folgenden.

Unmittelbar aus den Lehren der COVID-19-Krise lässt sich eine gestärkte EU-Agentur für öffentliche Gesundheit ableiten. Unter anderen sollte diese in die Qualifikationen des Gesundheitspersonals investieren und dann ihren flexiblen Einsatz in Notfällen erleichtern. Gleichzeitig sollte sie – wie auch schon von den europäischen Entscheidungsträgern angedacht – die Versorgung mit lebenswichtigen Medikamenten sicherstellen. Der rasche Ausbau einer modernen Verkehrsinfrastruktur erscheint geeignet, die oben genannten Ziele zu erreichen. Wir legen kalkulierte Vorschläge für zwei ehrgeizige Verkehrsinitiativen vor: ein eigenes europäisches Hochgeschwindigkeitsbahnnetz (Ultra-Rapid-Train) mit vier Strecken, die die Reisezeiten zwischen den Hauptstädten und Regionen der EU verkürzen und den innereuropäischen Flugverkehr drastisch reduzieren würde. Alternativ schlagen wir eine integrierte Europäische Seidenstraße vor, die Verkehrsträger nach chinesischem Vorbild kombiniert, und die EU mit Osteuropa verbindet, auch um die EU-Nachbarschaft besser einzubinden und dieser eine attraktive Entwicklungsperspektive anzubieten.

Im Bereich Energie/Entkarbonisierung liegt vielleicht die zentrale Herausforderung darin, den Green Deal zu „elektrisieren“. Europäische Finanzmittel sollten dafür eingesetzt werden, die Realisierung eines intelligenten und integrierten Stromnetzes für die Übertragung von 100 % erneuerbarer Energie (e-Highway) zu beschleunigen. Zusätzlich könnten ergänzende Batterie- und Öko-Wasserstoff-Projekte unterstützt werden. Nach dem Vorbild der SURE-Initiative könnte ein Programm zur Kofinanzierung geplanter Dekarbonisierungs- und Just-Transition-Politiken der Mitgliedstaaten aufgelegt werden. Das trägt der Tatsache Rechnung, dass erfolgreiche nationale Dekarboniserungsaktivitäten klare positive externe grenzüberschreitende Effekte haben. Die Finanzierung eines solchen Programms kann dem Modell des Kommissionsvorschlags folgen. Es gibt eine kaum zu befriedigende Nachfrage nach längerfristigen sicheren Anleihen, die auch die Durchführung der Geldpolitik erleichtern und die internationale Rolle des Euro stärken würden. Sinnvoll wäre die Schuldentilgung über neue gemeinsame EU-Ressourcen – idealerweise klimapolitisch wirksam (wie z.B. eine $CO_2$-Abgabe). Nutzergebühren könnte beispielsweise bei den Verkehrsinfrastrukturmaßnahmen eine Rolle spielen. Ein solches Programm wäre ein sichtbares Zeichen an alle Bürger*innen und alle Unternehmen und auch ans Ausland, dass Europa sein Schicksal selbst und gemeinsam in die Hand nimmt und die kurz- und längerfristigen Herausforderungen annehmen will.

Dr. Andrew Watt ist Referatsleiter für Europäische Wirtschaftspolitik am Institut für Makroökonomie und Konjunkturforschung (IMK) der Hans-Böckler-Stiftung.

Dr. Mario Holzner ist Geschäftsführer des Wiener Instituts für Internationale Wirtschaftsvergleiche (wiiw).

